# Breast Implant Capsule: Are You Going to Leave It in?

**DOI:** 10.1093/asjof/ojac005

**Published:** 2022-01-29

**Authors:** Stefano Rosso, Stefano Avvedimento, Elisa Grella, Antonio Guastafierro

We read with interest the article by Adam T. Hauch et al entitled “Subpectoral Implant Repositioning With Partial Capsule Preservation: Treating the Long-Term Complications of Subglandular Breast Augmentation.” ^1^ The demand for revision mastoplasty has increased over the past 10 years,^2^ and the surgeon’s decision on breast implant capsule management is still a matter of controversy. There is a consensus on the need to excise a malignant and severely contracted implant capsule completely. However, some critical questions remain: Can removal be avoided in cases where the capsule has a benign appearance? The utilization of residual implant capsules can be considered a safe procedure in the long run?

Over the years, implant capsules may significantly modify due to various factors that include inflammation, hematoma, seroma, subclinical infection, and radiation.^2^ In addition, the differentiation between benign and malignant capsule masses^2,3^ could be a diagnostic challenge. In our opinion, it is better to perform a complete capsulectomy in cases of implants removal and pocket conversion when the capsule has no role in supporting the new implants. To support our conclusion, we describe a particular case in which 2 capsular residues left in the breast simulated suspected masses, leading to diagnostic uncertainty and a new surgery for the patient. A 45-year-old patient with bilateral breast implants and with no family history of breast cancer presented to our attention in August 2021 with a palpable mass at the upper pole of the left breast. The patient had bilateral breast augmentation in 2010 and a second surgery in January 2015 to treat a capsule contracture. No pharmacological treatment was reported. From September 2015, the patient reported a right breast seroma that was transcutaneously aspirated and treated with corticosteroids. From October 2020, the patient noticed a painful swelling at the upper pole of the right breast. In April 2021, breast magnetic resonance imaging (MRI) with contrast reported “a hyperintense oval formation in T1 with a maximum size of 50 × 37 × 27 mm without enhancement after the injection of intravenous contrast at the upper inner quadrant of the right breast.”

On September 2021, a breast ultrasound showed a “coarse formation with mixed echotexture, fluid in the central component and with hyperechogenic calcific peripheral formations with a diameter of 60 mm” at the right upper pole of the right breast and a “solid formation with a diameter of about 45 mm with coarse, hyperreflective, calcified clods” at the left breast. Both formations appeared indissociable from the periprosthetic capsules.

**Figure 1.**
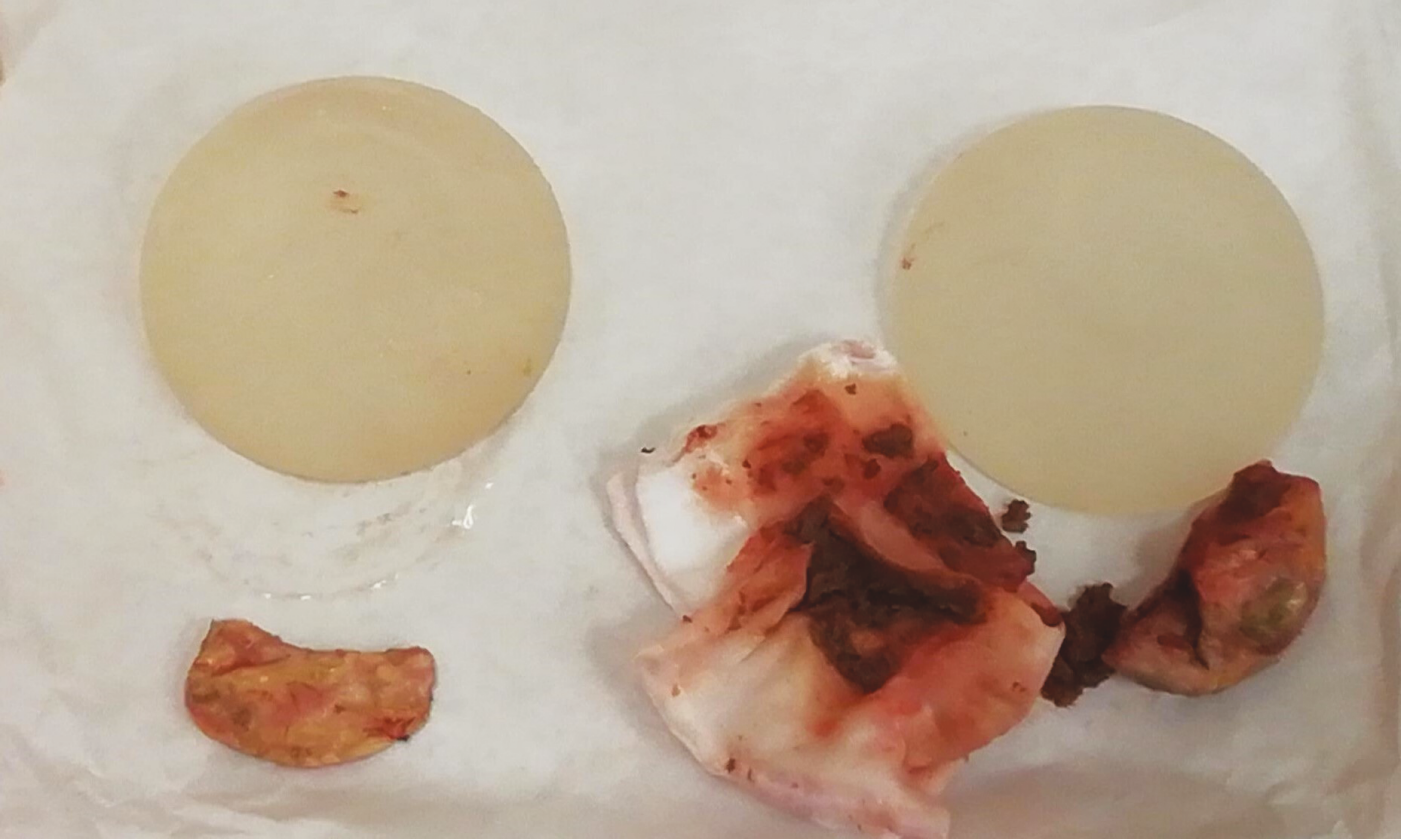
On the right, the capsulated formation filled with hematoma-like material that we found in the prepectoral plane of the right breast. On the left, the calcific formation is similar to a “cuttlefish bone” that we found attached to the pectoralis fascia in the prepectoral plane of the left breast. The 2 prosthetic implants appeared intact and without signs of deterioration.

Therefore, in September 2021, the patient underwent revisional surgery. We performed bilateral breast implant replacement and removal of the 2 capsular masses. Both implants were in a dual plane pocket, and the capsules did not look macroscopically pathological. On the right breast, in the prepectoral space firmly attached to the pectoralis fascia was found the presence of a capsulated formation filled with hematoma-like material. We found a calcific formation in the left breast similar to a “cuttlefish bone” attached to the pectoralis fascia in a prepectoral plane. We removed these 2 formations completely (Figure 1), and the intraoperative histological examination was negative for breast cancer. Then, we replaced the implants in a neosubpectoral pocket. Written informed consent for patient information and images was provided by a legally authorized representative. All the procedures performed in this study were in accordance with the 1964 Helsinki declaration and its later amendments or comparable ethical standards.

No complications occurred. The postoperative period was uneventful, and the patient was discharged from the hospital one day after surgery. Nahabedian^2^ asserted that thin capsules generally did not warrant excision because most would resorb over time, especially when subpectoral. However, even after several years of quiescence, an implant capsule can determine severe diagnostic dilemmas. We do not know why the previous surgeon preserved the old capsule in the prepectoral plane; perhaps, the capsular residue has been used as a pocket reinforcement just as Hauch et al^1^ described. In this case, it is probable that the old capsule, not excised at the time of implant replacement, showed a pseudo pathological behavior years after surgery. Preoperative differential diagnosis was challenging: US valuation showed 2 masses strongly adherent to the capsule that could simulate a bilateral anaplastic large-cell lymphoma (ALCL); even if the MRI showed no signs of not vascularization, a breast cancer diagnosis could not be excluded. Only the definitive histological examination clarified the diagnosis: “fibrosclerotic tissue with inflammatory monocytes and histiocytic infiltrate, the presence of macroprecipitates of calcium salts and cholesterin crystals, and the absence of malignant abnormalities.” Early reports from the 1990s suggested that capsulectomy following explantation was not always necessary.^2^ This was based on the fact that malignant or abnormal findings were rare; however, the surgeon should make the ultimate decision as to whether or not to remove it.^2,4^ In our case, the patient underwent surgery to obtain a definitive histological diagnosis of the 2 masses because the diagnostic imaging could not rule out any malignancy. This led to an increase in tangible and intangible costs for the patient, with additional surgical risks. Capsulectomy is considered the gold standard for the treatment of pathological capsules.^5^ It allows the restoration of the anatomical planes of the breast, removal of the biofilm, and the elimination of intracapsular vacuum space that can result in fluid and hematoma accumulation.^5^

In the literature, there is no clear evidence on the role of capsulectomy at the time of implant removal; Nahabedian asserted that thin capsules generally did not warrant excision because the majority would resorb over time, especially when subpectoral, but how far can we predict their behavior?^2^ Luckily in our case, there was a pseudo pathological transformation of the capsules without a malignant degeneration. There is still too much uncertainty about the behavior of the periprosthetic breast capsules over time concerning the types of implanted prostheses. Nowadays, we are discovering new diseases such as ALCL on patients with breast implants implanted up to 30 years ago. Goldberg et al^3^ showed the case of a squamous cell carcinoma arising in breast implant capsules that led to the patient’s death. The paucity of data on the behavior of periprosthetic capsules of the new smooth or microtextured implants implies adopting the precautionary principle. According to our experience, not all the capsules left in the breast undergo resorption and indeed can lead to diagnostic challenges and unnecessary secondary surgeries surgical. Since sufficient scientific information is not fully available on this topic, we recommend performing total capsulectomy in every situation of implants removal or pocket conversion, especially when the capsule is not needed for structural support of the pocket.
